# Ageism in COVID-19-Related Media Coverage: Examining Publications During the First Month of the Pandemic

**DOI:** 10.1093/geroni/igaa057.3421

**Published:** 2020-12-16

**Authors:** Mijin Jeong, Sarah Jen, Hyun Kang, Michael Riquino

**Affiliations:** 1 University of Kansas School of Social Welfare, Lawrence, Kansas, United States; 2 University of Kansas, Lawrence, Kansas, United States

## Abstract

The media has consistently described older adults as the population most vulnerable to COVID-19. Anti-ageism critics have taken issue with the oft-repeated statement that “only” older adults are at risk, a construction that dismisses and devalues the nuances within this population. The purpose of this study was to analyze instances of ageism in national media sources during the first month of the COVID-19 pandemic. A systematic search returned 287 articles concerning older adults and COVID-19 published in four major newspapers in the United States—USA Today, The New York Times, Los Angeles Times, and The Washington Post—between March 11 and April 10, 2020. Combining the strengths of content analysis and critical discourse analysis, we deductively and inductively reviewed the articles for patterns related to implicit and explicit forms of ageism. While ageism was rarely discussed explicitly, ageist bias was evident in implicit reporting patterns, such as frequent use of the phrase the elderly, which was often paired with statements describing older adults as vulnerable. Infection and death rates among older adults, as well as institutionalized care practices, were among the most commonly reported topics, providing a limited portrait of aging during the pandemic. While some authors utilized a survivor narrative by portraying older adults as having survived hardships, this construction implicitly places blame on those unable to do so. Older adults, when quoted directly, produced more complex and nuanced narratives of aging during the COVID-19 pandemic. Such narratives can combat societal ageism and promote self-determination and -definition.

